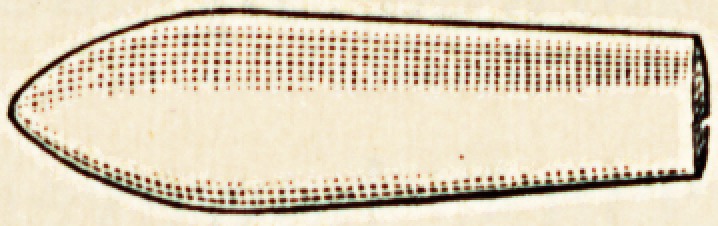# Notes on Preparations for the Sick

**Published:** 1904-06

**Authors:** 


### Notes on Preparations for the Sick.

**Tabloids: Veronal,** a new hypnotic.—Burroughs, Wellcome & Co., London.

This is a synthetic compound, chemically described as di-ethyl-malonyl-urea. It occurs in colourless crystals of a slightly bitter taste, and is soluble in 145 parts of water at 20° C. (68° F.). Its therapeutic action is somewhat similar to that of trional, but in some cases it appears to be preferable to that agent. Doses of 0.5 gramme to 1 gramme have been found to induce refreshing sleep, and the drug appears to be well tolerated by most patients. Its action, however, is in some cases very marked, and it is always well to begin with a ½-gramme dose until the susceptibility of the patient is ascertained.

"Tabloid" Veronal will be found a most accurate and convenient means of administering the drug.

One great advantage it has over other hypnotics in common use is, that it acts not only pleasantly but *quickly*, and in smaller dose (grs. 7 to 15). If the tabloid form is not desirable it may be dissolved in a cup of warm tea or other fluid.

---

**"Kepler" Malt Extract with Hæmoglobin.**—Burroughs, Wellcome & Co., London.

The direct administration of hæmoglobin has been followed by such favourable results, that this method of treatment has become established. As a blood-former hæmoglobin has been proved to be extremely efficacious. Containing, as it does, iron in organic combination precisely as it exists in the body, its assimilation is rapidly followed by definite improvement in health and strength.

It not unfrequently occurs, however, that patients evince a strong objection to the ordinary fluid, and in such cases "Kepler" Malt Extract with Hæmoglobin will be found satisfactory. It is efficient, stable and palatable, and possesses several marked advantages. In this preparation hæmoglobin is presented in a condition which ensures its immediate assimilation without disturbance of the digestive functions.

The value of "Kepler" Malt Extract is unquestionable.

Prepared from the finest winter-malted barley, the "Kepler" Extract contains the true malt diastatic ferment, and is rich in maltose, phosphates and albuminoids. This concentrated nutrient, combined with hæmoglobin, forms an admirable preparation for administration in anæmia, chlorosis, general debility, wasting diseases generally, and also in convalescence after illness.

"Kepler" Malt Extract with Hæmoglobin may be taken by adults in doses of from one teaspoonful to one tablespoonful, either alone or mixed with water or milk, after food, twice or thrice daily. The doses for children are proportionate to age.

---

**Manganese and Iron Citrate with Quinine, and Manganese and Iron Citrate with Strychnine.**—Burroughs, Wellcome & Co., London.

Scale preparations of manganese and iron citrate are now issued in combination with quinine and with strychnine respectively, both being readily soluble in water.

"Wellcome" Brand Manganese and Iron Citrate with Quinine contains about 7 per cent. of manganese, 14 per cent. of iron, and 15 per cent. of quinine. It may therefore be administered in doses of 3 to 10 grains with or after food.

"Wellcome" Brand Manganese and Iron Citrate with Strychnine contains about 7 per cent. of manganese, 14 per cent. of iron, and 1 per cent. of strychnine. Dose, 1 to 3 grains with or after food.

---

**"Enule" Brand Rectal Suppositories.**—Burroughs, Wellcome & Co., London.

"Enule" Suppositories obviate all the objectionable features of those hitherto in use, and provide a series of accurately-dosed suppositories, perfect in shape, in promptness of action, in therapeutic value, in keeping qualities, and in convenience.

They are produced by an improved process which ensures the even diffusion of the medicament throughout each suppository and exactitude of dosage. The novel shape adopted facilitates insertion and renders expulsion almost impossible.

Each suppository is enclosed in a sheath of pure tin-foil, which keeps it free from contamination and septic influences until the moment when it is required for use. The sheath is then easily stripped off, and the suppository inserted in the ordinary way. This sheath also enables "Enule" Suppositories to resist atmospheric changes to a degree hitherto impossible.

All the agents ordinarily employed in rectal medication are issued as "Enule" products.

Recent additions to the list:—

"**Enule" Quassin (Amorphous)**, gr. $\frac{1}{2}$.—A successful anthelmintic for the ascaris lumbricoides. Sustained treatment is necessary. One "Enule" product is administered on each of at least twelve successive nights.

"**Enule" Soap Compound.**—Each contains curd soap, gr. 7, and dried sulphate of sodium, gr. 7. This combination is used for the relief of constipation.

---

**Palatinoids.**—Oppenheimer, Son & Co., London.

**Creasote, Eucalyptol, and Iodoform.**—This combination should be very useful in the treatment of chronic cases of lung disease. Each palatinoid contains half a minim of creasote and one-tenth of a grain of iodoform. The dose is so small that the palatinoids may be increased in number and taken frequently.

**Ferrous Carbonate c̄ Manganese Dioxide.**—This is a combination of 2 grains of the latter with 4 grains of Blaud's pill compound. It is a combination which appears to be rather popular at the present time, more particularly in cases of menstrual disorders. The manganese dioxide, when given in pill form or as compressed tablets, is exceedingly difficult to dissolve, and hence the special utility of the palatinoid preparation.

---

**Hydrated Magnesia.**—Giles, Schacht & Co., Clifton.

We have received this sample of an old preparation of magnesia, which has recently been revived under the new designations of Milk of Magnesia and Maglactis. As an antacid and slight laxative, and as a sedative in cases of gastric catarrh, it should have attained a greater popularity than it has yet achieved under its newer designations.

---

**Angier's Petroleum Emulsion.**—Angier Chemical Company, London.

This emulsion has obtained a degree of popularity and apparently it has passed beyond the experimental stage and has an established reputation; but a recent analysis (Oliver Davis, University College Laboratory) shows that it contains 32.1 per cent. of a mineral oil, which is non-saponifiable and non-oxidisable. We fail to see how this indestructible oil can be of any use as nutriment in the alimentary canal, where the oxidising agents are surely not capable of doing more than can be done in the laboratory of the chemist. Perhaps the emulsion may have some mechanical and antiseptic action in the intestine, and although patients are understood to gain weight by its use, we cannot look upon it as in any way a substitute for cod-liver oil.

**Pepto-albuminoid, or Meat Biscuits.**—STEELE & MARSH, Bath.

The special advantage claimed for these biscuits is, that they are a complete food in themselves, containing concentrated the peptonised products of fresh meat in combination with extract of malt, pancreatised hydrocarbon, and a small dose of iron. They should be of great value to convalescents, to consumptives, diabetics, to delicate and anæmic children, as also to aged people. When nourishment is required in the night, and no nurse is available, these biscuits should be very convenient, and they are by no means unpleasant to take.

---

**Glycerol Heroin Hydrochloride Co.**—EVANS, GADD & CO., Bristol.

This glycerine solution of the heroin salt is combined with the active principles of senega root, wild cherry bark, and balsam of tolu.

Each fluid drachm contains one-sixteenth of a grain of the heroin hydrochloride.

It should be a comforting and valuable preparation to those who have irritating and obstinate affections of the respiratory tract.

---

**Betul-Ol; Methyl-Oleo-Salicylate.**—ANGLO-AMERICAN AND CONTINENTAL PHARMACEUTICAL COMPANY, Croydon.

A compound liniment of methyl salicylate distilled from the bark of the *Betula lenta*. It is absorbed readily by the skin, and is equivalent, weight for weight, to salicylate of soda. It is intended for local application in acute rheumatic and gouty affections, to supplement the action of other drugs given internally. It may be used with gentle friction or applied with a camel's-hair brush to the affected part, which should then be covered with oiled silk and cotton wool.

---

**Sanatogen.**—SANATOGEN COMPANY, London.

This powder, manufactured by Bauer & Cie, Berlin, is said to be "the best known tonic for the nerves and muscles." An analysis made by Mr. Oliver Davis, at the University College Laboratory, showed the presence of 93 per cent. of proteid matter, and the ash contained phosphates. It is therefore a highly nutritious powder, which should have a large range of utility. Professor Ewald, of Berlin, has written on its value in enteric fever. It is readily taken suspended in cold water or dissolved in any hot fluid, and a teaspoonful three times a day is usually considered to be a sufficient dose.

---

**Vittel (Vosges).**—GALLAIS & CO., London.

The mineral water of the "Grande Source" contains sulphates of calcium, magnesium and sodium, with some bicarbonates of lime, magnesia and soda, carbonic acid, and lithium carbonate. The total solids amount to 1.65 grammes per litre. Its special utility is in the renal excretion on the gouty diathesis. It is an excellent table water, more especially applicable to the gouty.

In an address by Dr. Landouzy, he remarks that "Vittel *at home* will become as easy as Vichy *at home*, and its reputation will be equal to the services it has already rendered and those it will render in the future. . . . The Vittel cure is a cure by drinking water that modifies metabolism, specially acting against the gouty diathesis, chronic joint trouble, and functional urinary or bilious calculosis."

## Figures and Tables

**Figure f1:**